# Sero-prevalence and risk factors of hepatitis B virus and human immunodeficiency virus infection among pregnant women in Bahir Dar city, Northwest Ethiopia: a cross sectional study

**DOI:** 10.1186/1471-2334-14-118

**Published:** 2014-03-01

**Authors:** Yohannes Zenebe, Wondemagegn Mulu, Mulat Yimer, Bayeh Abera

**Affiliations:** 1 Department of Microbiology, Immunology and Parasitology, College of Medicine and Health Sciences, Bahir Dar University, Bahir Dar, Ethiopia

**Keywords:** HBV, HIV, Pregnancy, Ethiopia

## Abstract

**Background:**

Hepatitis B virus (HBV) and human immunodeficiency virus (HIV) are the two most important agents of infectious diseases. Both HBV and HIV share common modes of transmission and have serious effects on both pregnant women and infants. In Bahir Dar city administration, there is a scarcity of information on sero-prevalence of HIV and HBV infection among pregnant women. The main objective of this study was to assess sero-prevalence and risk factors of HIV and HBV infection among pregnant women attending antenatal care in Bahir Dar city, Northwest Ethiopia.

**Methods:**

A cross-sectional study was conducted from March 2013 to April 2013. Socio-demographic and explanatory variables were collected using a structured questionnaire by face to face interview. Hepatitis B surface antigen (HBsAg) was detected using an enzyme linked immunosorbent assay (ELISA). HIV infection was also detected using the national HIV test algorithms. The results were analyzed with descriptive statistics and binary logistic regression. The odds ratio and 95% Confidence intervals were calculated.

**Results:**

A total of 318 pregnant women with the mean age of 25.72 (SD. ±5.14) years old were enrolled. Overall, 21/318 (6.6%) and 12 /318 (3.8%) of the pregnant women were positive for HIV and HBsAg, respectively. Of these, HIV/HBV co-infection rate was 4 (19.0%). Previous history of blood transfusion (AOR = 3.7, 95% CI, 9.02-14.84), body tattooing (AOR = 5.7, 95% CI, 1.24-26.50), history of surgery (AOR = 11.1, 95% CI, 2.64-46.88) and unsafe injection (AOR = 5.6, 95% CI, 1.44-22.19) were significantly associated with HBV infection. Previous history of piercing with sharp materials (AOR = 3.0, 95% CI 1.17-7.80) and history of abortion (AOR = 6.6, 95% CI 2.50-17.71) were also statistically significant for HIV infection.

**Conclusions:**

This study indicates that HIV and HBV infections are important public health issues in our region that need to be addressed. All pregnant women need to be screened for both HIV and HBV infections during antenatal care. Furthermore, health education about modes of transmission of HIV and HBV has to be given.

## Background

Human immunodeficiency virus (HIV) and Hepatitis B virus (HBV) infections are the two most important infectious diseases throughout the world particularly in developing countries. Both HIV and HBV share common ways of transmission in humans, which accounts for the high frequency of HIV-HBV co-infections. HIV and HBV have common risk factors like injectable drug use and blood transfusion [1]. In Ethiopia, the overall of prevalence of HIV among pregnant women attending anti-natal care (ANC) was 5.3%. Moreover, the prevalence was higher in pregnant women from urban (9.5%) than rural areas (2.2%) [2].

Hepatitis B virus causes both acute and chronic hepatitis. The global prevalence of chronic HBV infection is clustered as high (≥8%), intermediate (2-7%) and low (<2%). Thus, Ethiopia belongs to the high prevalence category [3]. Chronic active hepatitis B virus infection results in cirrhosis and hepatocellular carcinoma [4,5]. The probability of becoming a chronic HBV carrier is inversely related to age at the time of infection. Neonatal HBV infection always leads to a chronic carrier state [6,7]. Chronic HBV infection at birth occurs in approximately 90% of infants born from mothers who are positive for hepatitis B surface antigen (HBsAg) and hepatitis B e-antigen (HBeAg) [8]. In addition, maternal transmission of HBV predisposes infected newborns to liver cirrhosis and hepatocellular carcinoma in young adulthood [9]. HBV infection in infancy usually leads to a chronic carrier and then leads to liver cirrhosis and hepatocellular carcinoma. Therefore, the prevention of vertical transmission of HBV is very important, [10].

Co-infection rate of hepatitis B virus and HIV is common which leads to increased morbidity and mortality as compared to HIV or HBV mono-infections [11,12]. The prevalence of HIV-HBV co-infection is reported as high as 10-20% in countries where HBV infection is either endemic or intermediate to high [11]. Moreover, in countries where the viruses are highly endemic, the rate can be as high as 20-25% [11,13]. Both HIV and HBV are transmitted via sexual intercourse and mothers to offsprings. Thus, the prevalence of HIV and HBV infection in pregnant women can represent the majorities of the population in the communities because pregnant women are the interface for their sexual partners and infants.

Studies in Ethiopia have indicated that HBV and HIV are endemic with regional variation [14-16]. However, there is a scarcity of information on the prevalence of HBV and HIV infection among pregnant women in the study area. The present study was conducted to assess the sero-prevalence of HBV and HIV infection among pregnant women and to identify risk factors associated with HBV and HIV infection in Bahir Dar city.

## Methods

### Study area, period and design

A cross-sectional study was carried out from March 2013 to April 2013. The study was conducted in Bahir Dar administrative city. Bahir Dar is the capital of the Amhara National Regional State. The city is situated on the southern shore of Lake Tana, the source of the Blue Nile. It is located approximately 578 km Northwest of Addis Ababa, having a latitude and longitude of 11°36′N 37°23′E Coordinates and an elevation of 1840 meters above sea level. Based on the 2007 census conducted by the Central Statistical Agency of Ethiopia (CSA), Bahir Dar Special Zone has a total population of 221,991. Of these, 51.14% and 81.16% of them are females and urban dwellers respectively (18). In the city, there is one governmental referral hospital and ten health centers. The study participants were enrolled from the hospital and three randomly selected health centers.

### Sample size determination and sampling technique

The study population was all pregnant women who attended antenatal care from the selected hospital and health centers during a data collection time. The single population proportion formula was used to determine the sample size by considering 7.3% prevalence of HBV [14], 95% CI and a 3% margin of error. Accordingly, a total of 318 study participants was included. The number of participants in each of the selected health institutions was allocated proportionally based on the previous registered annual number of clients and a systematic random sampling technique was used to select the study participants.

### Data collection

A semi-structured questionnaire was used to collect socio-demographic and other explanatory variables of the study participants by face to face interview.

### Laboratory investigation

#### Detection of HBsAg

The presence of hepatitis B surface antigens (HBsAg) in serum was detected using EILSA (Linear chemicals. Joaquim Costa, Barcelona, Spain) according to the manufacturer’s instructions.

#### HIV screening

HIV infection among pregnant women was determined by anti-HIV antibody test (rapid test currently used in national algorithm for Ethiopia). KHB (Shanghai Kehua Bio-engineering Co., Ltd. China) was used for the first screening and positive samples were re-tested with STAT-PACK (Chembio HIV 1/2 STATPAK™ Assay, CHEMBIO DIAGNOSTIC SYSTEMS, INC., MEDFORD, NY, USA). Samples giving discordant results in the two tests were re-examined using tie-breaker, (Uni-Gold HIV, Trinity Biotech PLC, Wicklow, Ireland).

#### Quality control

To make sure that the questionnaire was appropriate and understandable, it was pretested on 30 pregnant women at the health center other than the actual study sites. The collected data were checked daily for consistency and accuracy. Standardized procedures were strictly followed during blood sample collection, storage and analytical process. Positive and negative controls were run alongside of the test.

#### Statistical analysis

Data was entered and analyzed using SPSS version 16.0. Binary logistic regression analysis was done to determine the association between explanatory variables and the outcome variables. All explanatory variables with a p-value less than or equal to 0.2 in the bivariate analysis were included in the multivariate logistic regression model to identify variables which were associated independently. Odds ratio (OR) and their 95% confidence intervals (CI) were calculated and the result was considered statistically significant at p < 0.05.

#### Ethical considerations

The study was conducted after obtaining ethical clearance from an ethical review committee of College of Medicine and Health Sciences, Bahir Dar University. Verbal consent was also secured from each study participant. HBsAg and HIV positive results were reported to physicians and/or health officers for treatment and any provision of antenatal care. Information on any course of the study was kept confidential. In addition, the clinical specimen collected during the study period was used for only the stated objectives.

## Results

### Socio-demographic characteristics

A total of 318 pregnant women took part in this study. Of whom, 214 (67.3%) were living in an urban setting. The mean age of the study participants was 25.72 (SD. ±5.14) years old. The majority of the study participants were housewives (56.9%) followed by merchants (13.2%) and governmental employees (11.0%). One hundred twenty five (39.3%) of the participants did not attend formal education. The majorities (93.1%) of participants were married.

### Prevalence of HIV and HBV infection

Overall, 21 (6.6%) and 12 (3.8%) of the pregnant women were HIV and HBsAg positive, respectively. Of these, 4 (19.0%) of pregnant women had both HBV/HIV co-infections. All HBsAg positive participants were among orthodox Christian followers. Prevalence of HBV and HIV did not vary significantly by age, residence, occupation, educational level, marital status, pregnancy trimester and income (Table 1).

**Table 1:** Prevalence of HBV and HIV among pregnant women attending antenatal care at Bahir Dar city, 2013 (n = 318)

**Socio-demographic variables**	**HBsAg**		**HIV status**	
	**Positive N (%)**	**Negative N (%)**	**Positive N (%)**	**Negative N (%)**
**Residence**				
Urban	7 (3.3)	207 (96.7)	14 (6.5)	200 (93.5)
Rural	5 (4.5)	99 (95.2)	7 (6.7)	97 (93.3)
**Age groups (yrs.)**				
**<21**	4 (4.9)	77 (95.1)	3 (3.7)	78 (96.3)
21-24	3 (3.6)	81 (96.4)	7 (8.3)	77 (91.7)
25-30	4 (3.7)	105 (96.3)	8 (7.3)	101 (92.7)
>30	1 (2.3)	43 (97.7)	3 (6.8)	41 (93.2)
**Religion**				
Orthodox	12 (4.4)	262 (95.6)	19 (6.9)	255 (93.1)
Muslim	0 (0.0)	40 (100.0)	2 (5.0)	38 (95.0)
Protestant	0 (0.0)	4 (100.0)	0 (0.0)	4 (100.0)
**Occupation**				
Merchant	1 (2.4)	41 (97.6)	3 (7.1)	39 (92.9)
Student	2 (11.1)	16 (88.9)	2 (11.1)	16 (88.9)
Housewife	6 (3.3)	175 (96.7)	10 (5.5)	171 (94.5)
Day laborer	2 (12.5)	14 (87.5)	2 (12.5)	14 (87.5)
Employer	0 (0.0)	35 (100.0)	3 (8.6)	32 (91.4)
Others	1 (3.8)	25 (96.2)	1 (3.8)	25 (96.2)
**Educational status**				
Not literate	6 (4.8)	119 (95.2)	5 (4.0)	120 (96.0)
Primary level	4 (5.1)	75 (94.9)	10 (12.7)	69 (87.3)
Secondary level	1 (1.7)	58 (98.3)	2 (3.4)	57 (96.2)
Technique level	0 (0.0)	7 (100.0)	2 (28.6)	5 (71.4)
Diploma	1 (3.3)	29 (96.7)	1 (3.3)	29 (96.7)
Degree and above	0 (0.0)	18 (100.0)	1 (5.6)	17 (94.4)
**Marital status**				
Unmarried	1 (6.7)	14 (93.3)	0 (0.0)	14 (100)
Married	9 (3.0)	287 (97)	19 (6.4)	277 (93.6)
Divorced	1 (16.7)	5 (83.3)	2 (33.3)	4 (72.7)
Widowed	1 (100.0)	0 (0.0)	0 (0.0)	1 (100.0)
**Average monthly income (Birr)**				
<1000	7 (5.9)	112 (94.1)	8 (6.7)	111 (93.3)
1000-1500	3 (4.7)	61 (95.3)	5 (7.8)	59 (92.2)
1501-2300	0 (0.0)	58 (100.0)	4 (6.9)	54 (93.1)
>2300	2 (2.6)	75 (97.4)	4 (5.2)	73 (94.8)
**Pregnancy stage**				
1° trimester	0 (0.0)	60 (100.0)	4 (6.7)	56 (93.3)
2° trimester	8 (5.7)	133 (94.3)	9 (6.4)	132 (93.6)
3° trimester	4 (3.5)	113 (96.5)	8 (6.8)	109 (93.2)

### Associated risk factors for hepatitis B virus and HIV infection

Multivariate logistic regression analysis was conducted to assess independent risk factors for HBV and/or HIV infections. History of blood transfusion (AOR = 3.70, 95% CI, 9.02-14.84), body tattooing (AOR = 5.74, 95% CI, 1.24-26.50), previous history of surgery (AOR = 11.1, 95% CI, 2.64-46.88) and unsafe injection (AOR = 5.65, 95% CI, 1.44-22.19) were statistically significant risk factors for HBV infections (Table 2). For HIV infection, previous history of piercing with sharp materials (AOR = 3.0, 95% CI 1.17-7.80) and history of abortion (AOR = 6.6, 95% CI 2.50-17.71) were statistically significant (Table 3).

**Table 2:** Association of explanatory variables and hepatitis B virus infection among pregnant women attending antenatal care at Bahir city, 2013 (n = 318)

**Variables**	**HBsAg**		**COR (CI)**	**AOR (CI)**	**P- value**
	**Positive N (%)**	**Negative N (%)**			
**History of blood transfusion**			8.8 (2.70-29.19)***	3.7 (9.02-14.84)	0.01
Yes	6 (16.2)	31 (83.8)			
No	6 (2.1)	275 (97.9)			
**History of tooth extraction**			7.2 (2.19-23.49)**	2.2 (0.46-10.08)	0.32
Yes	7 (12.3)	50 (87.7)			
No	5 (1.9)	256 (98.1)			
**Tooth tattooing**			8.4 (2.44-28.74)**	2.9 (0.67-12.70)	0.15
Yes	8 (11.9)	59 (88.1)			
No	4 (1.6)	247 (98.4)			
**Body tattooing**			5.1 (5.36-19.32)**	5.7 (1.24-26.50)	0.02
Yes	9 (7.4)	113 (92.6)			
No	3 (1.5)	193 (98.5)			
**History of surgery**			12.9 (3.84-43.37)***	11.1 (2.64-46.88)	0.001
Yes	6 (21.4)	22 (78.6)			
No	6 (2.1)	284 (97.9)			
**History of unsafe injection**			10.2 (3.07-33.73)***	5.6 (1.44-22.19)	0.01
Yes	7 (15.9)	37 (84.1)			
No	5 (1.8)	269 (98.2)			
**History of abortion**			6.6 (1.96-21.99)**	0.6 (0.08-4.29)	0.58
Yes	5 (14.3)	30 (85.7)			
No	7 (2.5)	306 (96.2)			
**HIV/AIDS status**			8.5 (2.33-31.07**	0.4 (0.57-2.19)	0.26
Positive	4 (19.0)	17 (81.0)			
Negative	8 (2.7)	289 (97.3)			

**Key**: COR (crude odds ratio), AOR (adjusted odds ratio).

***P-value <0.001, **0.05 < p-value >0.001.

**Table 3:** Association of explanatory variables with HIV infection among pregnant women attending antenatal care at Bahir Dar city, 2013 (n = 318)

**Variables**	**HIV status**		**COR (CI)**	**AOR (CI)**	**P- value**
	**Positive N (%)**	**Negative N (%)**			
**History of piercing with sharp materials**			3.7 (1.52-9.24)**	3.0 (1.17-7.80)	0.02
Yes	10 (14.7)	58 (85.3)			
No	11 (4.4)	239 (95.6)			
**History of tooth extraction**			1.9 (0.71-5.21)*	0.5 (0.14-1.99)	0.35
Yes	6 (10.5)	51(89.5)			
No	15 (5.7)	246 (94.3)			
**Tooth tattooing**			3.1 (1.24-7.68)**	1.4 (0.51-4.32)	0.47
Yes	9 (13.4)	58 (86.6)			
No	12 (4.8)	239 (95.2)			
**History of surgery**			6.5(2.39-18.04)***	2.5 (0.76-8.67)	0.12
Yes	7 (25.0)	21 (75.0)			
No	14 (4.8)	276 (95.2)			
**History of abortion**			7.8 (3.01-20.28)***	6.6 (2.50-17.71)	0.001
Yes	9 (25.7)	58 (74.3)			
No	12 (4.2)	271 (95.8)			
**Ear pricing**			2.9 (0.83-10.11)*	1.9 (0.51-7.06)	0.33
Positive	18 (8.3)	200 (91.7)			
Negative	3 (3.0)	97 (97.0)			

**Key**: COR (crude odds ratio), AOR (adjusted odds ratio).

***P-value <0.001, **0.05 < p-value >0.001, *0.2 ≤ p-value >0.05.

## Discussion

In the present study, the overall prevalence of HIV infection among pregnant women was higher (6.6%) than the national HIV prevalence among pregnant women (5.3%) [2]. Likewise, HIV prevalence was higher than the study conducted in Southern Ethiopia (1.8%) [17] and Mali, (0.4%) [18]. In contrast, it is lower than reports from Gondar in Ethiopia [14] and in Nigeria (8.4%) [19]. The majority of HIV positive participants in this study were among the age group 21–24 years old. Similarly, 13% of HIV prevalence was reported in the same age group (21–24 years) in Gondar, Ethiopia [14]. This age group is the most vulnerable group for HIV infection.

According to the WHO classification, the prevalence of HBV among pregnant women in this study was intermediate (2-7%) [3]. The prevalence of HBV infection in the present study conforms with reports from the study in Nigeria (3.8%) [19] and Jimma, Ethiopia (3.7%) [15]. However, the prevalence of HBV in the current study was higher than the study conducted in India (0.9%) [20] and Tripoli, Libya (1.5%) [21]. In contrast, it was lower than the study documented in Taiwan (15.5%) [22], Keffi in Nigeria (6.67%) [23] and Gondar in Ethiopia (7.3%) [14]. These differences might be attributable to cultural and behavioral differences for the risk factors of HBV infection.

The frequency of HBV and HIV co-infection (19.0%) in this study was higher than the study done in Southern Ethiopia (0.6%) [17] and Nigeria (9.5%) [19]. Furthermore, the prevalence of HIV infection among HBsAg positive pregnant women was 33.3%. Most of the time comorbidity of HBV and HIV infection is common among risk groups. Because, both HBV and HIV share common mode of transmission.

Regarding socio-demographic status of the study participants, the highest proportion of HBV infection was among pregnant women who were not literate and those whose average monthly income was less than one thousand Ethiopian Birr. Likewise, similar finding was also reported from Jimma in Ethiopia [15]. The highest prevalence of HBsAg was detected in pregnant women who were on a secondary trimester. This was also in line with the study conducted in Minna, Nigeria [24].

In this study all of the socio-demographic variables were not statistically significant. However, pregnant women who had a previous history of blood transfusion were about 3.7 times more likely to be positive for HBsAg (AOR = 3.70, CI, 9.02-14.84). A similar study, which was conducted in Egypt identified this variable as significant associated factor [25]. Study participants who have body tattooing on any part of their body was also 5.7 times more likely to be HBsAg positive (AOR = 5.74 CI, 1.24-26.50). Studies conducted from Bamako, Mali and Addis Ababa, Ethiopia identified that body tattooing has a significant association with HBV infection [16,26]. Moreover, surgical application was one of the risk factor which was statistically significant, (AOR = 11.1 CI, 2.64-46.88) [25].

Unsafe injection like traditional healers’ practice is one of the risk factors for HBV transmission. In our study, pregnant women who had a history of unsafe injection were 5.6 times more likely to be HBV positive (AOR = 5.6 CI, 1.44-22.19). Previous studies showed that patients who received more injections and injected with non-sterile needle were more likely to be infected with hepatitis virus [27]. This practice is also common in our country where different cultural application on unsafe injection is practiced. Among explanatory variables, previous history of piercing with sharp materials (AOR = 3.0, 95% CI 1.17-7.80) and history of abortion (AOR = 6.6, 95% CI 2.50-17.71) were significantly associated with HIV infection.

Detection of other markers of HBV like HBeAg and DNA was not possible because of lack of laboratory setup. Moreover, HIV/RNA viral load was not determined for HIV positive pregnant women. These can be considered as the major limitation of this study.

## Conclusions

This study showed that HIV and HBV infections are important public health issues in our region that need to be addressed. All pregnant women need to be screened for both HIV and HBV infection during antenatal care. Blood transfusion, body tattooing, surgery and unsafe injection were risk factors for HBV infection. Moreover, previous history of piercing with sharp materials and history of abortion were major risk factors for HIV infection. Thus, health education about these risk factors in particular and the mode of transmissions and prevention of HIV and HBV in general should be given.

## Competing interests

The authors declare that they have no any competing interest.

## Authors’ contributions

YZ: Contributed from inception of the research question to design, analysis, interpretation and preparation of the manuscript. BA: contributed to proposal development, data analysis and manuscript writing, WM, MY: participated in proposal development, analyzed the data, edit and write the manuscript. All authors read and approved the final manuscript for publication.

## Pre-publication history

The pre-publication history for this paper can be accessed here:

http://www.biomedcentral.com/1471-2334/14/118/prepub
